# Comprehensive collection of uniaxial stress-strain data for rubberized concrete

**DOI:** 10.1016/j.dib.2024.111189

**Published:** 2024-12-03

**Authors:** Abdulaziz Alsaif

**Affiliations:** Civil Engineering Department, College of Engineering, King Saud University, P. O. Box 800, Riyadh 11421, Saudi Arabia

**Keywords:** Waste tires rubber concrete, Stress-strain curves, Uniaxial compression test, Recycled tires

## Abstract

This dataset article encompasses a thorough compilation of 80 uniaxial stress-strain datasets obtained from cylindrical rubberized concrete specimens subjected to compression testing. Data collection was meticulously conducted through a systematic review and extraction of stress-strain datasets from 68 rubberized concrete mixtures sourced from diverse literature references, incorporating rubber of different origins, sizes, volumes and characteristics. Additionally, stress-strain data for 48 cylindrical specimens, representing 12 different mixes with various rubber sizes and contents, were obtained from laboratory experiments performed by the author. The datasets provide valuable insights for researchers interested in the compressive behavior of rubberized concrete and offers valuable resources for further analysis and modeling studies.

Specifications Table

**Table:** 

Subject	Civil and Structural Engineering
Specific subject area	Uniaxial compressive stress-strain data generated for rubberized concrete
Type of data	▪ Raw data ▪ Graphs (uniaxial stress-strain curves)
Data collection	The data collection was sourced from published papers using the web-plot-digitizer tool to extract data from stress-strain plot images. Additionally, uniaxial compression tests were performed on cylindrical rubberized concrete specimens to produce stress-strain data. Stress-strain data from a total of 68 rubberized concrete mixes were compiled from published reports, along with stress-strain data from laboratory tests on 48 cylindrical specimens representing 12 different rubberized concrete mixes.
Data source location	Civil Engineering Department, College of Engineering, King Saud University, P. O. Box 800, Riyadh 11421, Saudi Arabia
Data accessibility	Repository name: Mendeley Data Data identification number: (or DOI or persistent identifier): 10.17632/8dx7ghgsvv.1 Direct URL to data: https://data.mendeley.com/datasets/8dx7ghgsvv/1
Related research article	Y.M. Abbas, A. Alsaif, Enhanced nonlinear models for critical compressive stress-strain characteristics of rubberized concrete: comprehensive experimental data and robust evaluation methodology, Construction and Building Materials. 433 (2024) 136691 https://doi.org/10.1016/j.conbuildmat.2024.136691

## 1 Value of the Data

• The comprehensive collection of uniaxial stress-strain data for cylindrical rubberized concrete specimens offers valuable insights into the mechanical behavior of rubberized concrete under compression.
• Researchers can use these data to explore how different rubber replacement proportions affect the stress-strain behavior of rubberized concrete, facilitating comparative studies and analyses.
• The dataset serves as a reference for validating analytical and numerical models, as well as simulations developed to predict the mechanical characteristics of concrete containing different rubber sizes and contents, enhancing the accuracy and reliability of future computational analyses in this field.
• Accessing these stress-strain data will enable researchers to explore inventive design approaches for rubberized concrete structures, optimizing mix compositions and design parameters to enhance performance and durability while reducing environmental impact.
• The dataset can be utilized to enhance the prediction of safety factors through reliability studies. The comprehensive range of stress-strain data provides a detailed analysis of the performance variability of rubberized concrete, which is essential for determining accurate safety factors.
• The dataset is valuable for machine learning studies focused on predicting the behavior of rubberized concrete. The large and diverse dataset is well-suited for training machine learning models to forecast various performance aspects of rubberized concrete under different conditions.

## 2 Background

Many studies in the literature have assessed the use of recycled tire rubber particles (RTRP) to replace varying proportions of natural fine and/or coarse aggregates in the preparation of concrete mixes. It has been consistently demonstrated that the inclusion of RTRP in concrete leads to a reduction in its strength and stiffness. The extent of this effect is influenced by the content and size of the RTRP incorporated [[1], [2], [3]]. Despite these drawbacks, the inclusion of RTRP in concrete mixtures in fact improves a number of concrete properties, such as energy absorption and dissipation capacity, ductility and toughness [4]. Hence, understanding the behavior of rubberized concrete mixtures that were manufactured with different rubber particle sizes and contents and under various stress-strain conditions is important for optimizing its performance in structural applications and ensuring its reliability. The rationale behind compiling this dataset includes constructing a comprehensive understanding of the uniaxial compression behavior of rubberized concrete specimens incorporating RTRP of various sources, sizes, volumes and attributes, and also providing useful information for stress-strain analytical models in future studies.

## 3 Data Description

The raw stress-strain data for rubberized concrete mixtures were collected and organized in a file named “Stress-Strain Data for Rubberized Concrete” using Mendeley Data, a cloud-based communal repository, ensuring secure storage and easy access. The datasets are meticulously organized to aid researchers interested in examining the uniaxial compressive behavior of rubberized concrete mixtures, cast with different RTRP sizes and contents, ensuring both accessibility and interpretability. The file comprises three sheets, each with its own description:

1. Own data sheet: This sheet contains stress-strain data collected from laboratory experiments and tests performed by the author on cylindrical rubberized concrete specimens. The stress-strain data were generated through uniaxial compression tests on specimens prepared with various RTRP sizes and contents. Each dataset within this sheet corresponds to an average curve computed from the tested rubberized concrete mixtures.
2. Literature-based data sheet: This sheet contains raw stress-strain data extracted from published papers using the web-plot-digitizer tool [5]. All stress-strain data in this sheet correspond to the average stress-strain curve for a rubberized concrete mixture obtained from different literature sources.
3. References sheet: This sheet provides references to the papers from which the dataset in the “Literature-based data” sheet was extracted.

**Fig. 1:**
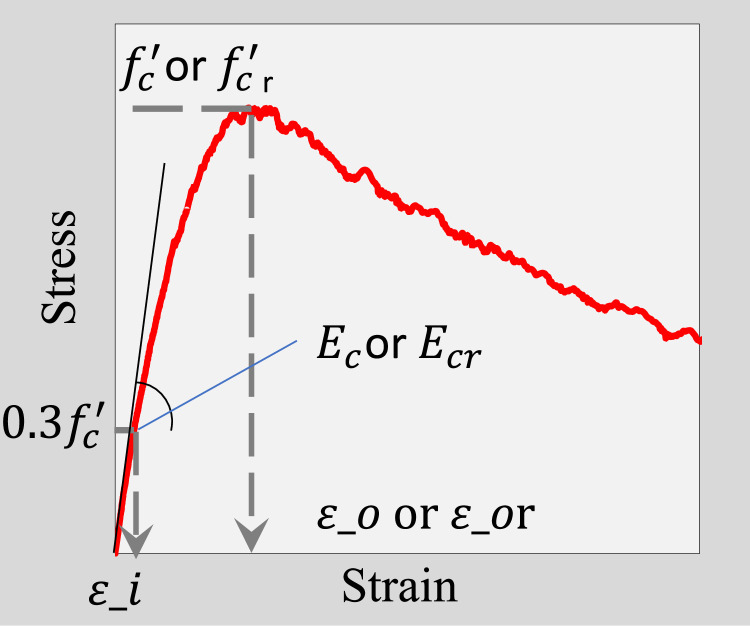
Definition of the key parameters of the stress-strain curve.

Table 1 presents information about the rubberized concrete mixtures contained in the above file, including the name(s) of the author(s), mix ID, specimen size, and volume percentage of fine and/or coarse aggregate replaced by RTRP. Table 2 provides detailed information on the standard methods used to produce and test the specimens, including loading speed, equipment for measuring strain and stress, capping procedures, specimen curing age. Table 3 presents the key parameters of the compressive stress-strain behavior of rubberized aggregate concrete mixes, including peak stress (${f^{\prime }}_{c}\;\text{or}\;{f^{\prime }}_{cr}$), strain at peak stress ($\varepsilon_{o}\;\text{or}\;\varepsilon_{or}$), and the modulus of elasticity ($E_{c}\;\text{or}\;E_{cr}$), as defined in Fig. 1.

**Table 1:** Information on rubberized concrete mixtures.

Authors	Refs.	Mix ID	Specimen size	% of fine RTRP	% of coarse RTRP
			mm	By volume	By volume
Author data		M1	ϕ100 × 200	0	0
		M2		0	20
		M3		0	40
		M4		0	0
		M5		50	0
		M6		0	0
		M7		20	20
		M8		40	40
		M9		0	0
		M10		20	20
		M11		0	0
		M12		30	30
Bompa et al.	[2]	M13	ϕ100 × 200	0	0
		M14		20	20
		M15		40	40
		M16		60	60
Batayneh et al.	[6]	M17	ϕ150 × 300	0	0
		M18		20	0
		M19		40	0
		M20		60	0
		M21		80	0
Moustafa et al.	[7]	M22	ϕ150 × 300	0	0
		M23		10	0
		M24		20	0
		M25		30	0
Noaman et al.	[8]	M26	ϕ100 × 200	0	0
		M27		5	0
		M28		10	0
		M29		15	0
Li et al.	[9]	M30	ϕ100 × 200	0	0
		M31		6	0
		M32		12	0
		M33		18	0
Raffoul et al.	[1]	M34	ϕ100 × 200	0	0
		M35		10	0
		M36		20	0
		M37		100	0
		M38		0	10
		M39		0	20
		M40		0	40
		M41		0	60
		M42		0	100
		M43		40	40
		M44		60	60
Eldin and Senouci	[10]	M45	ϕ150 × 300	0	0
		M46		100	0
		M47		0	100
Strukar et al.	[11]	M48	ϕ150 × 300	0	0
		M49		10	0
		M50		20	0
		M51		30	0
		M52		40	0
Wu et al.	[12]	M53	ϕ150 × 300	0	0
		M54		0	10
		M55		0	15
		M56		0	20
		M57		0	30
		M58		0	40
		M59		0	50
		M60		0	80
		M61		0	100
Alsaif et al.	[3]	M62	ϕ100 × 200	0	0
		M63		20	20
		M64		40	40
		M65		60	60
Abyaneh et al.	[13]	M66	ϕ150 × 300	0	0
		M67		0	5
		M68		0	10
		M69		0	15
Elnaggar et al.	[14]	M70	ϕ150 × 300	0	0
		M71		0	10
		M72		0	20
		M73		0	30
		M74		0	40
		M75		0	50
		M76		0	60
		M77		0	70
		M78		0	80
		M79		0	90
		M80		0	100

**Table 2:** Detailed parameters for specimen production, testing, and measurement.

Authors	Refs.	Standard method used to produce specimens	Standard method used to test specimens	Loading speed	Equipment used to measure stress-strain	Capping procedure	Age of moist curing
Author data		ASTM C192	ASTM C39	0.35 mm/min	Universal Testing Machine with a maximum load capacity of 3000 kN	Ends of cylindrical specimens were ground and capped with sulfate mortar	28 days
Bompa et al.	[2]	Not specified	Not specified	0.1 mm/min	Stiff four-post Instron Satec with a maximum load capacity of 3500 kN machine	Ends of cylindrical specimens were ground. & The rubberized concrete cylinders were capped with high strength mortar and further polished with sand paper	28 days
Batayneh et al.	[6]	ASTM C192	ASTM C39	Not specified	Universal Testing Machine with a maximum load capacity of 300 kN	Not specified	28 days
Moustafa et al.	[7]	ASTM C192	Not specified	0.2 mm/min.	MTS machine	Ends of cylindrical specimens were ground	56 days
Noaman et al.	[8]	ASTM C192	ASTM C39	0.3 N/mm^2^/s	Hydraulic machine	Not specified	28 days
Li et al.	[9]	AS 1012.2 & AS 1012.8.1	Not specified	0.001 mm/s.	Uniaxial Baldwin compression machine	Ends of cylindrical specimens were ground	28 days
Raffoul et al.	[1]	Not specified	Not specified	0.25 MPa/s & 0.1 MPa/s for cylinders with very high rubber contents (above 60% fine or coarse replacement)	Universal Testing Machine with a maximum load capacity of 3000 kN	Ends of cylindrical specimens were confined using high-strength and high-ductility post tensioned metal straps of thickness 0.8 mm and width 13 mm	25 days
Eldin and Senouci	[10]	ASTM C 192	Not specified	Not specified	Not specified	Not specified	28 days
Strukar et al.	[11]	HRN EN 12390-1 HRN EN 12390-2	HRN EN 12390-13:2013	0.01 MPa/s.	Automatic Compression machine with a capacity of 2000 kN	Not specified	28 days
Wu et al.	[12]	ASTM C192	Not specified	0.3 mm/min	MTS machine with a capacity of 3000 kN	The top casting end of cylindrical specimens were capped with sulfur mortar	28 days
Alsaif et al.	[3]	EN 12390-2	EN 12390-3	0.3 mm/min	Universal Testing Machine with a maximum load capacity of 1000 kN	Ends of cylindrical specimens were ground	28 days
Abyaneh et al.	[13]	Not specified	Not specified	0.6 mm/min	Universal Testing Machine with a maximum load capacity of 2000 kN	Ends of cylindrical specimens were covered by a capping compound	28 days
Elnaggar et al.	[14]	Not specified	ASTM C39	0.5 mm/min	2000 kN Instron hydraulic machine	two ends of the cylinders are capped by placing them on melted sulfuric compounds	28 days.

**Table 3:** Key parameters of the compressive stress-strain curve of rubberized concrete [15].

Authors	Refs.	Mix ID	${f^{\prime }}_{c}\;\text{or}\;{f^{\prime }}_{cr}$	$\varepsilon_{o}\;\text{or}\;\varepsilon_{or}$	$\varepsilon_{i}$	$E_{c}\;\text{or}\;E_{cr}$
			MPa	µm/m	µm/m	GPa
Author data		M1	48.7	1705	390	38.1
		M2	32.1	1410	290	33.3
		M3	21.2	1390	290	22.4
		M4	78.2	2760	570	41.1
		M5	24.4	2010	350	21.1
		M6	83.2	2440	595	42.8
		M7	28.9	1685	330	26.4
		M8	9.7	1195	250	11.7
		M9	49.0	2265	435	33.6
		M10	27.4	2040	370	22.5
		M11	47.5	3700	630	22.9
		M12	14.8	1990	385	11.6
Bompa et al.	[2]	M13	70.9	2249.1	451.8	46.0
		M14	29.7	2093.3	527.3	18.5
		M15	13.2	1332.2	255.1	15.0
		M16	6.2	1232.3	160.1	10.2
Batayneh et al.	[6]	M17	27.5	2767.9	1113.1	6.6
		M18	17.6	2238.1	1059.5	4.3
		M19	10.2	1881	881	3.2
		M20	6.8	2023.8	738.1	2.4
		M21	3.4	1309.5	523.8	1.6
Moustafa et al.	[7]	M22	67.0	3010.8	1126.4	16.0
		M23	52.6	3075.8	1191.3	14.1
		M24	52.3	3216.6	1397.1	11.1
		M25	39.6	3205.8	1234.7	8.1
Noaman et al.	[8]	M26	41.3	879.5	399	35.1
		M27	35.0	902.9	373.1	24.9
		M28	33.1	1096	411	20.2
		M29	29.9	1141.8	479.3	14.3
Li et al.	[9]	M30	50.3	2573.1	584.8	28.3
		M31	44.9	1891.3	570.7	26.6
		M32	38.9	2380.3	368.4	32.4
		M33	35.2	2430.4	582.3	20.4
Raffoul et al.	[1]	M34	62.5	2165.7	568	38.3
		M35	55.0	1895.3	441.9	37.6
		M36	45.6	1837.2	395.3	34.0
		M37	9.7	1139.5	139.5	16.4
		M38	46.3	1810.7	343.2	37.6
		M39	38.4	1562.1	307.7	35.5
		M40	26.4	1656.8	331.4	25.0
		M41	9.7	1139.5	139.5	16.4
		M42	8.2	1076.9	189.3	37.6
		M43	10.5	1329	245.2	16.2
		M44	6.6	1161.3	206.5	10.6
Eldin and Senouci	[10]	M45	58.6	1861.8	––	32.8
		M46	17.3	1829	279.2	16.9
		M47	3.1	1122	––	5.4
Strukar et al.	[11]	M48	40.4	2871.8	522.1	18.8
		M49	23.1	3459.2	946.4	7.0
		M50	11.3	5221.4	1338	2.5
		M51	8.9	3720.3	1338	2.5
		M52	2.7	4993	1240.1	0.6
Wu et al.	[12]	M53	32.7	2237.7	416	27.4
		M54	24.8	2067.6	331.8	25.1
		M55	24.1	1951.2	341.5	24.5
		M56	24.2	2013.9	383.1	20.3
		M57	18.3	1824.7	483	14.3
		M58	15.0	2139.1	391.3	11.8
		M59	10.2	2019.2	445.8	6.7
		M60	24.2	2013.9	383.1	20.3
		M61	4.0	4047.3	1105.7	14.3
Alsaif et al.	[3]	M62	56.5	1630.9	392	44.3
		M63	38.6	2199.5	493.5	25.7
		M64	11.1	1285.7	291.8	13.7
		M65	6.4	1758	303.2	7.1
Abyaneh et al.	[13]	M66	64.2	2676.9	432.5	40.2
		M67	63.5	2228.2	591.2	35.7
		M68	57.1	2455.7	482.1	32.3
		M69	50.4	2087.7	443.1	29.0
Elnaggar et al.	[14]	M70	70.1	2137.7	460	47.0
		M71	49.1	2327.3	439.5	30.7
		M72	46.4	2624	516.5	26.5
		M73	43.2	2840.2	735.3	19.1
		M74	38.8	2872.6	696.6	18.5
		M75	30.6	2962.3	679.2	14.1
		M76	26.8	3198.5	728.2	12.2
		M77	43.2	2840.2	735.3	19.1
		M78	22.4	3880.6	1109.2	18.5
		M79	17.5	3636.5	732.2	8.0
		M80	14.7	3878.9	620	6.6

**Fig. 2:**
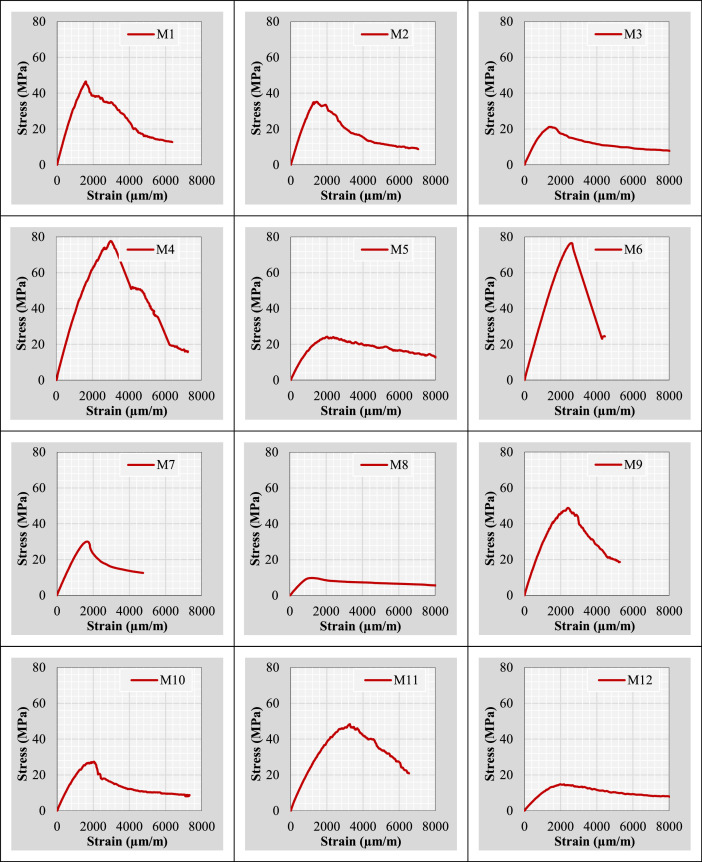

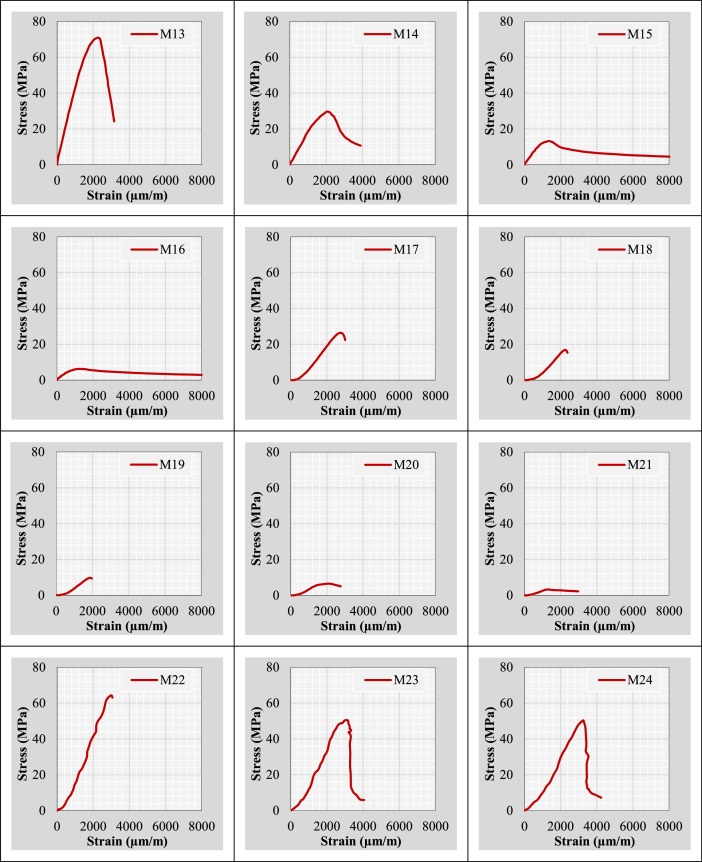

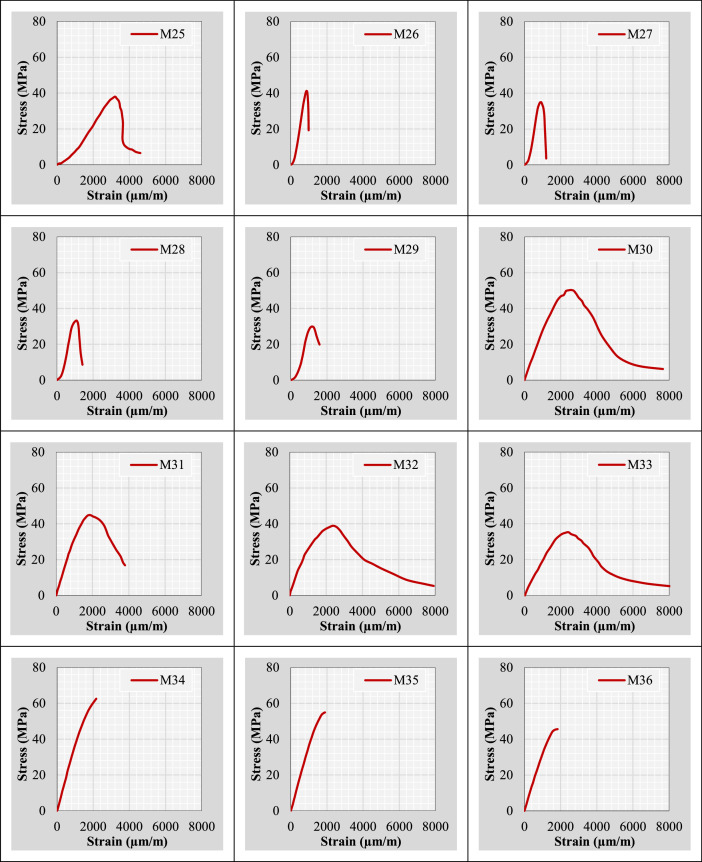

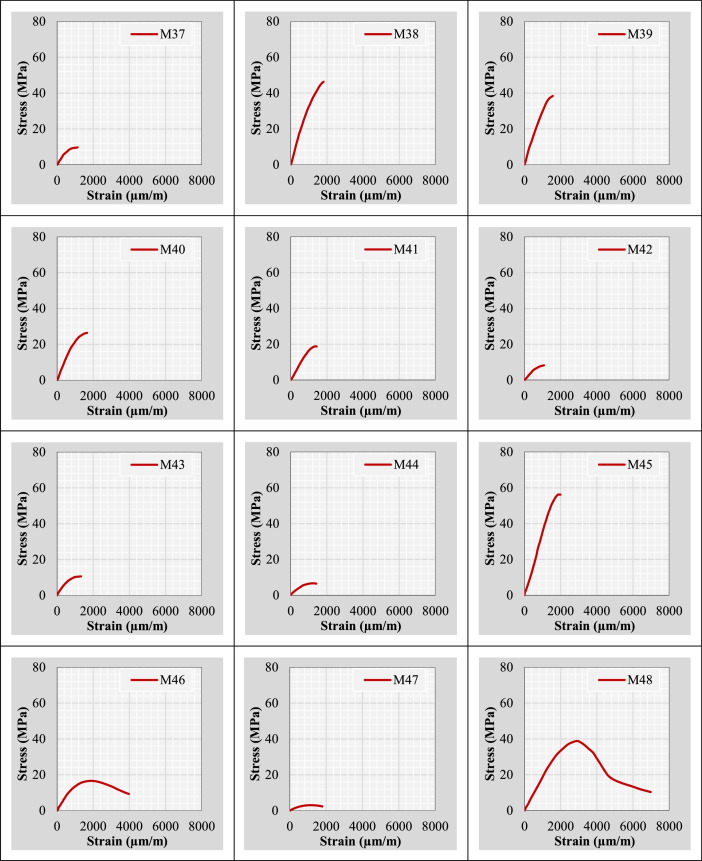

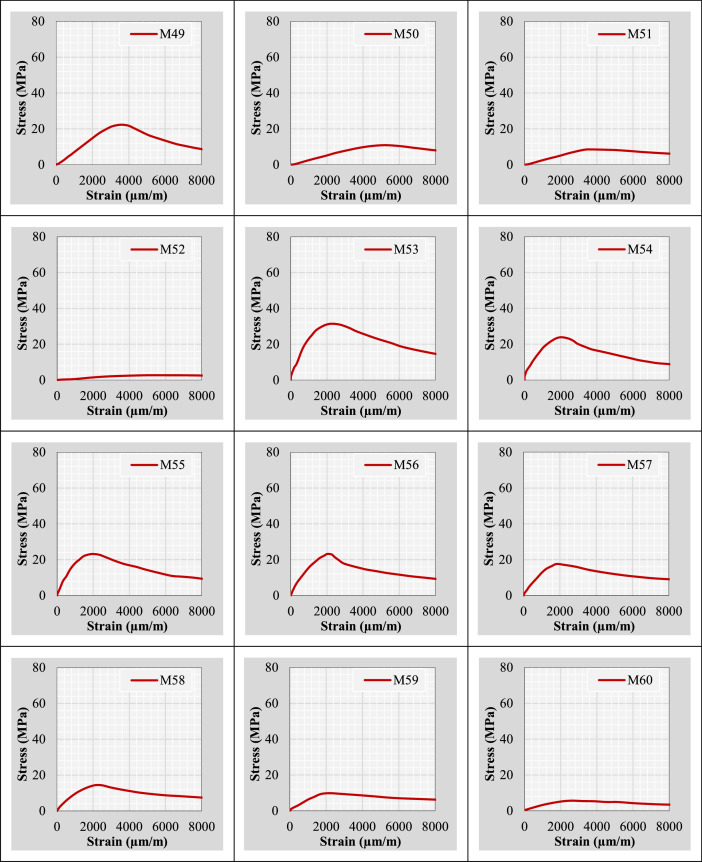

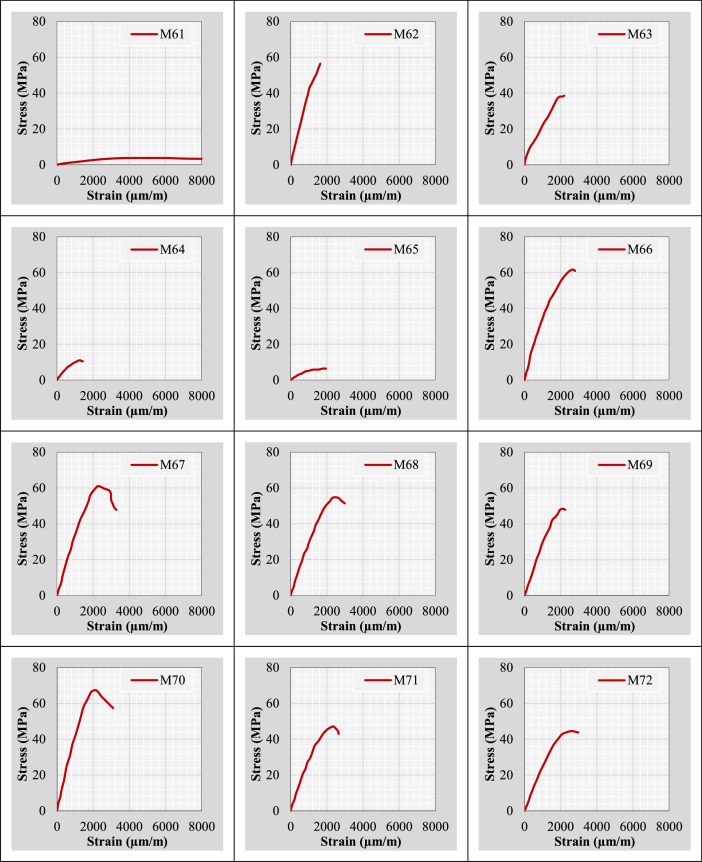

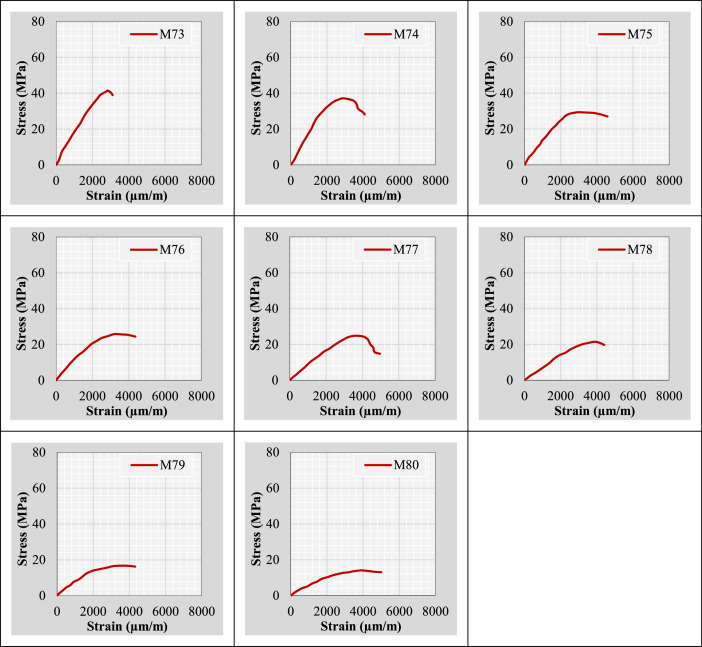
Stress-strain curves representing the extracted and tested rubberized concrete mixtures.

Continuing from the previous information, the author plotted stress-strain curves for all mix IDs using the datasets provided in the “Own data” and “Literature-based data” sheets. These curves are presented in Fig. 2.

## 4 Experimental Design, Materials and Methods

In this study, 12 different concrete mixes were prepared, resulting in the production of 48 cylindrical rubberized concrete specimens (four replicas for each mix). The following sections describe the materials and methods used for the preparation and testing of these cylindrical specimens under uniaxial compression loading.

Portland cement (PC) served as the main binder, with supplementary cementitious materials silica fume (SF) and pulverized fly ash (PFA) replacing 20% by weight of the cement (10% by weight allocated to SF and 10% by weight allocated to PFA).

**Fig. 3:**
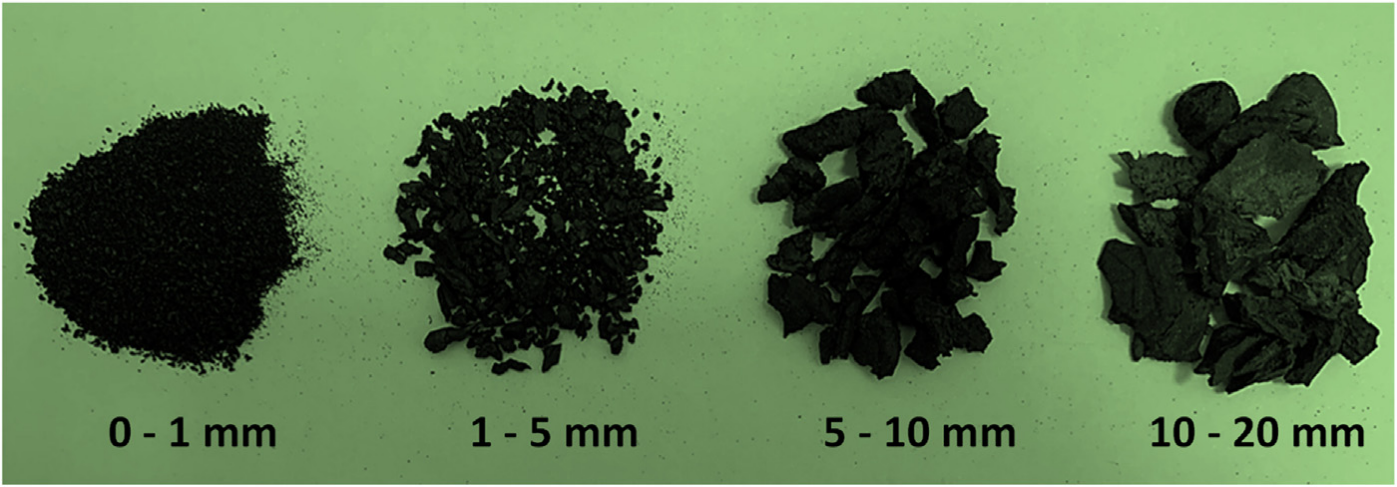
Photographs of fine and coarse RTRP used in this study.

**Fig. 4:**
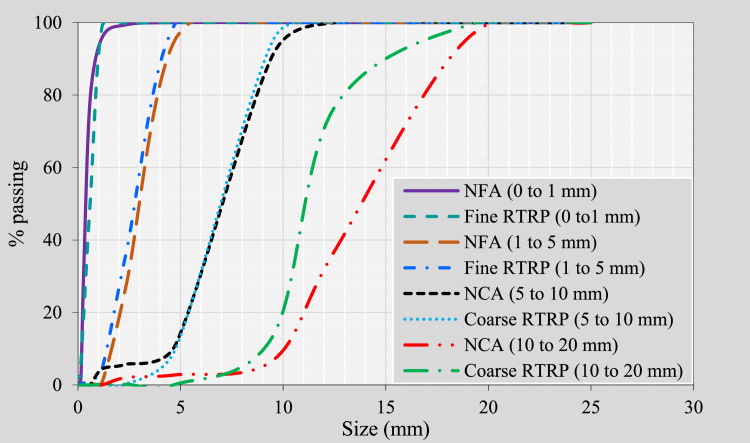
Particle size distributions of all aggregates.

Locally sourced materials were utilized for the manufacture of the concrete mixtures. Natural coarse aggregates (NCA) comprised crushed limestone rock in sizes ranging between 5–10 mm and 10–20 mm. These aggregates exhibited a bulk specific gravity (BSG) of 2.62, loose bulk density (LBD) of 1575 kg/m^3^, and water absorption (WA) of 2.0%. Natural fine aggregates (NFA) comprised sand and crushed limestone, with sizes of 0–1 mm and 1–5 mm, respectively. The sand exhibited a BSG of 2.65, LBD of 1605 kg/m^3^ and WA of 0.3%; the crushed limestone had a BSG of 2.65, LBD of 1650 kg/m^3^ and WA of 2.5%. A local supplier mechanically shredded the recycled tires into fine and coarse rubber particles of similar sizes to the NFA and NCA being replaced (see Fig. 3). These RTRP were then used to replace the NFA and NCA at a volumetric ratio of 1:1. The RTRP demonstrated a BSG of 0.82, LBD of 430 kg/m³ and WA of 1.3%. Fig. 4 shows the particle size distribution of all aggregates.

As shown in Table 4, the concrete mixtures were divided into five distinct groups, each with varying compositions of binder contents and percentages of RTRP replacing NFA and NCA.

• Group #1 consisted of Mixes M1 to M3, which were conventional concrete with a fixed Portland cement content of 350 kg/m³ and coarse RTRP replacing 0, 20 and 40% of NCA.
• Group #2 included Mixes M4 and M5, where similar binder contents were used, but fine RTRP replaced 0 and 50% of the NFA.
• Group #3 comprised Mixes M6 to M8, with a higher binder content including SF and PVA, and RTRP replacing 0, 20 and 40% of both NFA and NCA.
• Group #4 consisted of Mixes M9 and M10, with similar binder content to Group #1 but with RTRP replacing 0 and 20% of both NFA and NCA.
• Group #5 consisted of Mixes M11 and M12, with similar binder content to Group #1 but with RTRP replacing 0 and 30% of both NFA and NCA.

**Table 4:** Composition of rubberized concrete mixtures investigated.

Group #	#1			#2		#3			#4		#5	
Mix ID	M1	M2	M3	M4	M5	M6	M7	M8	M9	M10	M11	M12
PC kg/m^3^	350			350		360			350		350	
SF kg/m^3^	-			-		45			-		-	
PVA kg/m^3^	-			-		45			-		-	
NFA kg/m^3^	800			800	400	892	713.6	535.2	800	640	800	560
NCA kg/m^3^	1045	836	627	1045		870	696	522	1045	836	1045	731.5
Water L/m^3^	140			140		170			140		140	
Superplasticizer L/m^3^	2.7			2.4	3.3	10.1			3.5		3.5	
Fine RTRP (vol %)	-			-	50	-	20	40	-	20	-	30
Coarse RTRP (vol %)	-	20	40	-		-	20	40	-	20	-	30

These variations in binder contents and RTRP percentages and sizes allowed for a comprehensive investigation into the mechanical properties of rubberized concrete.

**Fig. 5:**
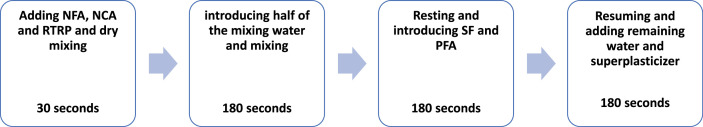
Sequence of concrete mixing.

The concrete ingredients were mixed in a 200 L pan mixer and then cast, compacted and cured based on ASTM C192 [16]. Fig. 5 shows the sequence of concrete mixing.

Upon completion of the mixing procedure for all concrete constituents, the workability of the concrete was evaluated, then it was cast into concrete cylinders (ϕ100 × 200 mm) and compacted. These cylinders were then wrapped in damp burlap to retain moisture for 24 h. The cylinders were then demolded and placed in water for 28 days before testing.

**Fig. 6:**
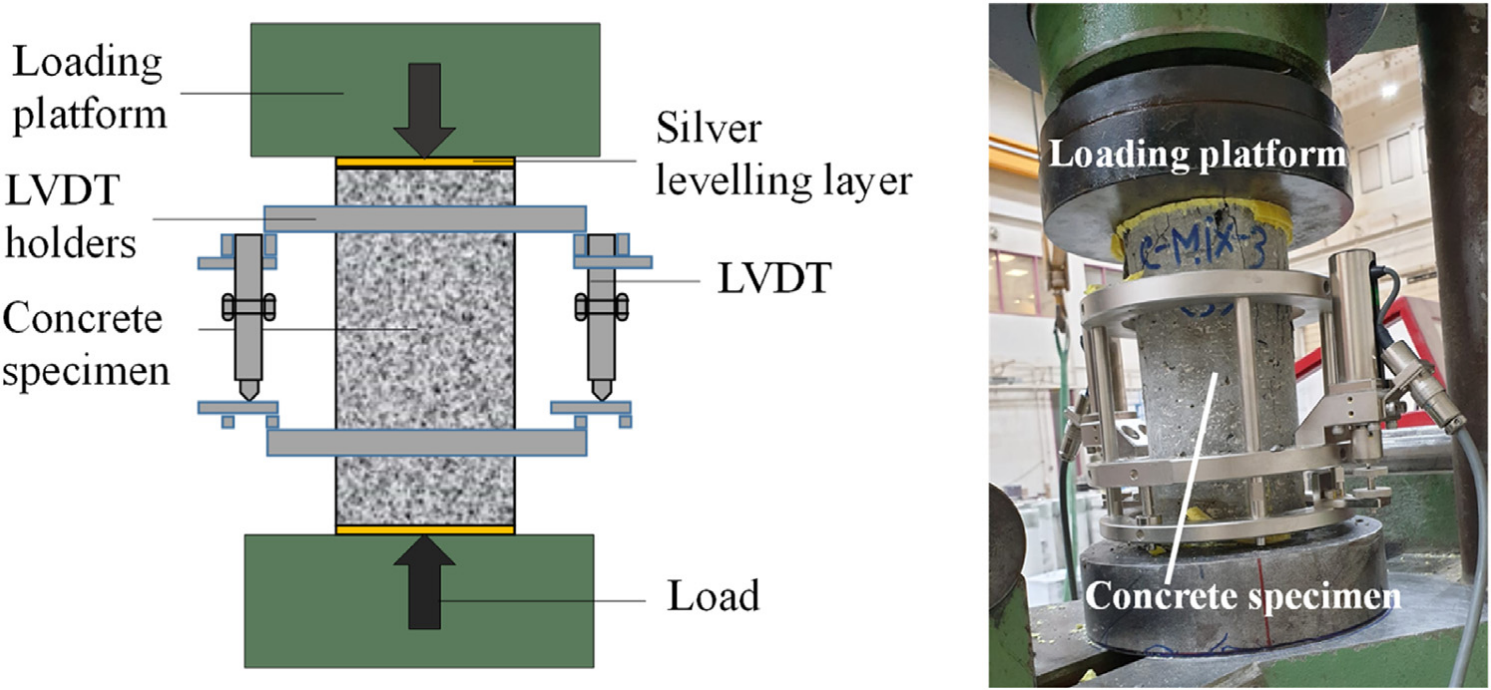
(*l*) Schematic diagram and (*r*) photograph of the uniaxial compression test.

The uniaxial stress-strain compressive behavior was analyzed for four replicate ϕ100 × 200 mm specimens from each rubberized concrete mix. A compressometer with two linear variable differential transformer (LVDTs) recorded the axial stress vs. strain changes in the specimen during loading (see Fig. 6).

**Fig. 7:**
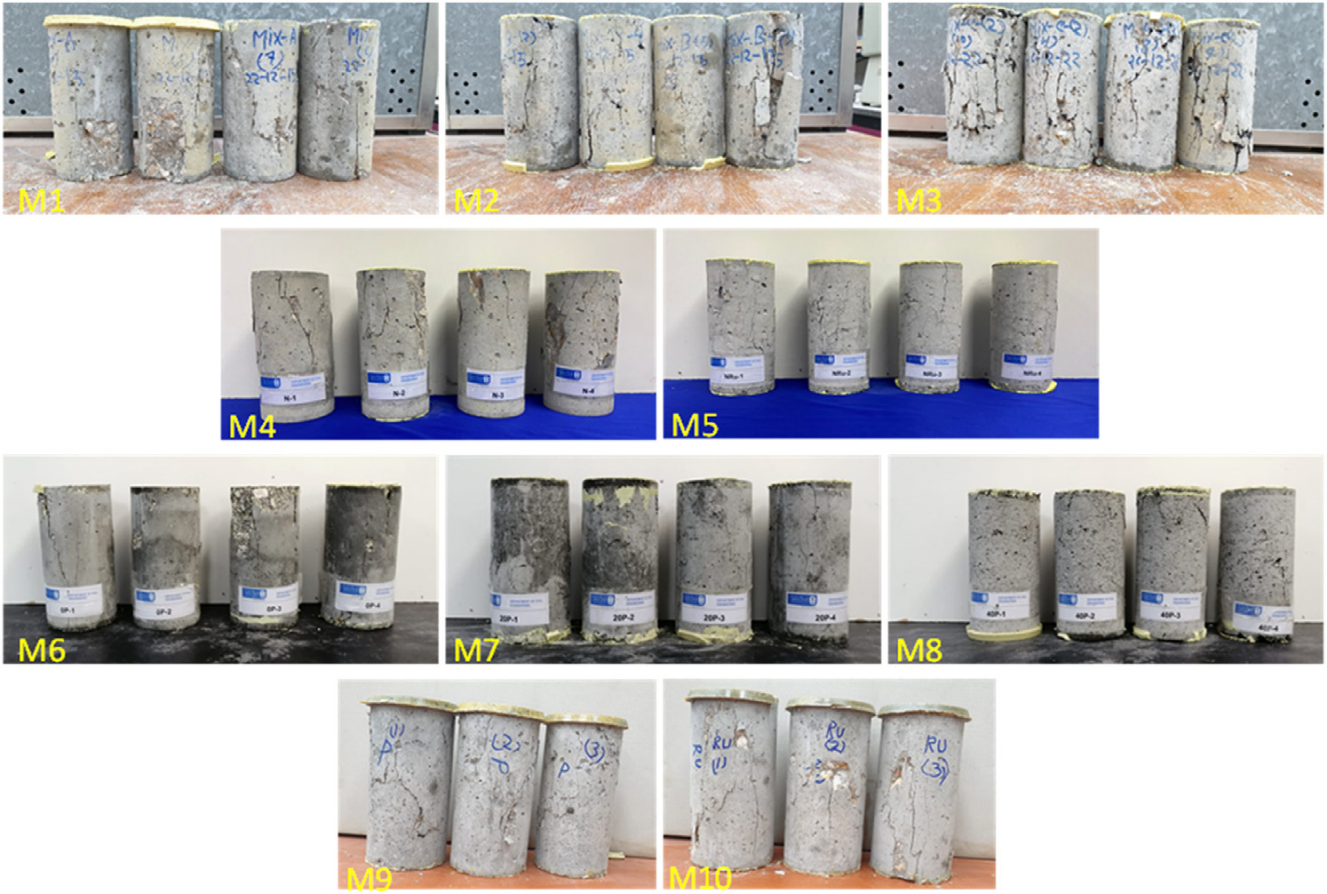
Crack development in concrete specimens at failure (specimens #M1–#M10).

The failure modes and cracking pattern of the concrete specimens of all mixes, except M11 and M12, after loading in axial compression are shown in Fig. 7.

## Limitations

While the dataset provided in this investigation yields valuable insights into the behavior of rubberized concrete under uniaxial loading, several limitations warrant acknowledgment. Primarily, the dependence on published literature for data aggregation raises concerns regarding potential inconsistencies or inaccuracies stemming from variations in casting and testing conditions. The variation in specimen sizes reported in literature sources may introduce potential effects due to differences in dimensions, even though the 2:1 aspect ratio is constant. Additionally, the utilization of the web-plot-digitizer tool for data extraction may have introduced inaccuracies or discrepancies in the numerical data obtained, thereby impacting the overall reliability of the dataset. Furthermore, the relatively limited number of studies and rubberized concrete formulations incorporated in the dataset may constrain the generalizability of the findings to broader contexts.

## Ethics Statement

The author hereby confirms that he has thoroughly reviewed and adhered to the ethical requirements for publication in Data in Brief. Specifically, it is affirmed that the current work does not involve human subjects, animal experiments, or the utilization of data collected from social media platforms.

## CRediT authorship contribution statement

**Abdulaziz Alsaif:** Conceptualization, Methodology, Data curation, Formal analysis, Software, Project administration, Supervision, Funding acquisition, Writing – original draft, Writing – review & editing.

## Data Availability

Mendeley DataStress-strain Data for Rubberized Concrete (Original data).
